# Post-earthquake Distress and Development of Emotional Expertise in Young Adults

**DOI:** 10.3389/fnbeh.2018.00091

**Published:** 2018-05-08

**Authors:** Francesca Pistoia, Massimiliano Conson, Antonio Carolei, Maria G. Dema, Alessandra Splendiani, Giuseppe Curcio, Simona Sacco

**Affiliations:** ^1^Department of Biotechnological and Applied Clinical Sciences, Neurological Institute, University of L’Aquila, L’Aquila, Italy; ^2^Neuropsychology Laboratory, Department of Psychology, University of Campania Luigi Vanvitelli, Caserta, Italy; ^3^Department of Biotechnological and Applied Clinical Sciences, University of L’Aquila, L’Aquila, Italy

**Keywords:** earthquake, anxiety, depression, emotional, expertise

## Abstract

After a natural disaster like an earthquake about 15% of the population experience a post-traumatic stress disorder (PTSD). However, even those without a diagnosis of PTSD can suffer from disorders of the affective sphere, including anxiety, depression and alteration of emotion recognition. The objective of this study was to investigate the neuropsychological and emotional profile of students living in the earthquake-affected areas of L’Aquila, Italy. A group of students living in L’Aquila at the time of the 2009 earthquake was recruited, and compared to a control group of students not living in any earthquake-affected areas. Participants were assessed by means of the Beck Depression Inventory (BDI) scale, the State-Trait Anxiety Inventory (STAI), the Insomnia Severity Index (ISI), the Intolerance of Uncertainty Scale Short Form, the Uncertainty Response Scale (URS), the Anxiety Sensitivity Index 3 (ASI-3), and the Eysenck Personality Questionnaire-Revised Short Form (EPQ-RS). Participants also took part in two behavioral experiments aimed at evaluating their ability to recognize facial expressions (by means of the Ekman and Friesen Pictures of Facial Affect) and to evaluate emotionally evocative scenes (by means of the International Affective Picture System (IAPS)). Results showed that students living in the earthquake-affected areas had a general increase of anxiety and anticipation of threats. Moreover, students living in the earthquake-affected areas showed a significantly higher overall accuracy in recognizing facial expressions as compared to controls. No significant differences between the two groups were detected in the evaluation of emotionally evocative scenes. The novel result lies in the greater accuracy of earthquake victims in recognizing facial expressions, despite the lack of differences from controls in evaluating affective evocative scenes. The trauma exposure may have increased vigilance for threats in earthquake victims, leading them to systematically pay attention to potential signs of approaching threats, such as emotional facial expressions, thus progressively developing particular “emotional expertise.”

## Introduction

After a natural disaster, like an earthquake, population has an increased vulnerability to developing psychological and psychiatric disorders. The most frequently reported is Post-Traumatic Stress Disorder (PTSD), which is a mental health problem triggered by life-threatening events (Neria et al., 2008). The Diagnostic and Statistical Manual of Mental Disorders (DSM-5) criteria for PTSD include the direct or indirect exposure to a terrifying event; the occurrence of at least one of five intrusion symptoms; the presence of an avoidance behavior concerning trauma-related stimuli and of negative alterations in cognitions and mood; and the occurrence of trauma-related alterations in arousal and reactivity. Moreover, in order to fulfill criteria for the PTSD diagnosis, symptoms are required to last for more than 1 month, to create distress or functional impairment in person affected, and to not be attributable to other illnesses or to the use of medications or substances (American Psychiatric Association, 2013). Prevalence rates of PTSD following an earthquake are extremely heterogeneous across studies and range from 4% to 67% (Tang et al., 2017). Factors accounting for this variability include the population studied, the age groups considered, the time elapsed since the traumatic event, the sample size and the study design. As mental health looks like a continuum, it is reasonable to expect that a population which has been exposed to a natural disaster cannot simply be split into two groups, one suffering from well-defined psychiatric disorders and the other enjoying optimal mental health. It is likely that a certain proportion of individuals belong to a gray zone where a psychiatric illness is not diagnosed, but the person is not experiencing complete mental well-being. This is to be expected even more in the young, in whom traumatic experiences may exert a profound impact on psychological and emotional behaviors. Such an impact may not necessarily manifest as impaired processing of emotions, but might rather lead to heightened sensitivity to specific emotional signals, especially those conveying self-relevant potential threatening information, as in the case of negative emotional facial expressions (Bell et al., 2017).

Defining the specific nature of these “compensative” emotional responses could have relevant translational implications, including the implementation of interventions aimed at encouraging the development of coping strategies after natural disasters. International guidelines recommend using cognitive-behavioral therapy (CBT) a few weeks after a disaster or other shocking events to reduce psychopathological symptoms, in particular those related to PTSD (Te Brake et al., 2009). A recent review of the literature has demonstrated that, among the cognitive-behavior therapy techniques, exposure techniques seem particularly effective for treating PTSD after earthquakes (Lopes et al., 2014). However, recent cognitive models of anxiety disorders consistently reported attentional biases toward threat-related stimuli not only in persons with a clinical condition but also in nonclinical individuals reporting high levels of anxiety (Dalgleish et al., 2003; Bar-Haim et al., 2007; MacLeod and Grafton, 2016). It is worth noting here, that together with PTSD, depression is one of the most common psychiatric disorders appearing in earthquake survivors several weeks or months after traumatic events, and often persisting for years (Yule, 2001). Interestingly, current views on depression underlined the central role of cognitive dysfunctions (Gonda et al., 2015), and especially of attentional biases, in the etiology and maintenance of the disorder (Disner et al., 2011). On this basis, clarifying the nature of the dysfunctional emotional processing in earthquake-exposed persons could allow choosing the most effective cognitive-behavioral technique allowing survivors to boost resilience and to acquire specific mental skills to manage threats.

In this venue, the objective of the present study was to investigate the neuropsychological and emotional profile of earthquake-exposed university students and to identify any specificity in the way they process affective information including emotional facial expressions and emotionally evocative scenes. Moving from previous evidence demonstrating an increased sensitivity to negative facial emotions in earthquake-exposed individuals as compared to non-exposed controls (Bell et al., 2017), here we aimed at assessing whether this enhanced response to visual affective stimuli was specific to facial expressions or, instead, it was related to a pervasive enhanced sensitivity towards all visual stimuli conveying affective information, as in the case of affective complex scenes. Thus, we compared earthquake-exposed students with non-exposed control students on recognition of facial expressions (by means of the Ekman and Friesen’s set of pictures) and on judgment of emotionally evocative scenes (by means of the International Affective Picture System (IAPS)).

Basing on previous literature reports, the following alternative findings may be expected: (i) earthquake-exposed and not exposed students may show a similar emotional profile without significant differences in processing emotional stimuli, whether these be facial expression or emotional evocative scenes; (ii) earthquake-exposed students may have developed an increased and generalized sensitivity to emotional information, resulting in increased accuracy in processing all kinds of emotional information, both emotional facial expressions and emotionally evocative scenes; and (iii) earthquake-exposed students may show a selective increased accuracy in recognizing emotional facial expression, despite an unchanged perception of their own internal emotional responses to evocative scenes not involving faces. The results of the present study might provide empirical evidence about the engagement of specific coping and resilience strategies in young adults after an environmental traumatic event.

## Materials and Methods

### Experimental Setting

The earthquake epicenter of L’Aquila (central Italy) was used as the experimental setting. On April 6th 2009, L’Aquila was hit by an earthquake lasting 20 s and with a magnitude of 6.3 on the moment magnitude scale. The earthquake caused 309 deaths and the destruction of the city, with 65,000 inhabitants being forced to leave their homes. The main earthquake was followed by thousands of aftershocks in the subsequent months with a severe psychological burden on the whole population. About 7 years after this devastating earthquake, with the city only partially rebuilt, the population experienced additional earthquakes (August and October 2016 and January 2017). Although not causing further destruction, these resulted in great psychological distress for the people of L’Aquila, who had not yet recovered from the psychological, social and economic consequences of the 2009 earthquake.

### Participants

A sample of students living in L’Aquila at the time of the 2009 earthquake was recruited for the participation in the study. To be included, participants had to fulfill the following criteria: age >18 years at the time of the inclusion; stable residence in L’Aquila at the time of the 2009 earthquake; no history of previous or coexistent neurological or psychiatric diseases, including PTSD, or assumption of drugs or substances acting on the central nervous system; and signed informed consent to participate in the study. A control group of students, matched by age and sex, not living in any earthquake-affected areas, was recruited and used for comparison: these participants were all psychologically healthy individuals without a personal or family history of mental illness. The whole sample included 107 students. Forty-eight subjects belonged to the experimental earthquake-exposed group (20 males and 28 females, mean age = 22.6, SD = 2.3 years) and 59 to the control group (30 males and 29 females, mean age = 23.1, SD = 1.6 years).

The research protocol was approved by the Internal Review Board of the University of L’Aquila (01/2017). The study was conducted in accordance with the ethical standards of the Helsinki Declaration and signed informed consent was obtained from all the participants.

### Procedures

#### Self-Reported Measures

All participants were assessed by means of the following formalized measures: the Beck Depression Inventory (BDI; Beck, 1967), the State-Trait Anxiety Inventory (STAI; Spielberger et al., 1983; Pedrabissi and Santiniello, 1989), the Insomnia Severity Index (ISI; Bastien et al., 2001; Castronovo et al., 2016), the Intolerance of Uncertainty Scale Short Form (IUS-12; Freeston et al., 1994), the Uncertainty Response Scale (URS; Greco and Roger, 2001), the Anxiety Sensitivity Index 3 (ASI-3; Taylor et al., 2007; Petrocchi et al., 2015) and the Eysenck Personality Questionnaire-Revised Short Form (EPQ-RS; Eysenck et al., 1985; Picconi et al., 2018).

The BDI (Beck, 1967) is a 21 items self-report inventory; it is one of the most widely used psychometric tests for the assessment of depression severity. Total score can range from 0 to 63, with higher scores indicating increasing level of depressive symptoms. The total score is usually used as dependent variable.

The STAI (Spielberger et al., 1983; Pedrabissi and Santiniello, 1989) is a commonly used measure of trait and state anxiety: here, the 20 items only for the assessment of trait anxiety have been used. Total score can range between 20 and 60, and also in this case high score reflect high level of anxiety. The total score has been considered as dependent variable.

The ISI (Bastien et al., 2001; Castronovo et al., 2016) is a 7-item self-report questionnaire assessing the nature, severity and impact of insomnia. The usual recall period is the “last month.” This tool allows evaluating different dimensions (sleep onset, sleep maintenance and early morning awakening problems, sleep dissatisfaction, interference with daytime functioning, noticeability of sleep problems by others, and distress caused by the sleep difficulties). A Likert scale is used to rate each item, yielding a total score ranging from 0 to 28, with higher scores indicating higher severity of insomnia symptoms. The total score has been taken into consideration as dependent variable.

The Tolerance of Uncertainty Scale Short Form (IUS-12; Freeston et al., 1994) is a short version of the original 27-item Intolerance of Uncertainty Scale that measures responses to uncertainty, ambiguous situations and the future. The 12 items are rated on a 5-point Likert scale and can provide a measure of both Prospective Anxiety and Inhibitory Anxiety, as well as a total measure of uncertainty (by summing the scores to all the 12 items). We considered the total score as dependent variable.

The URS (Greco and Roger, 2001) is a scale for the evaluation of styles of coping with uncertainty. The 48 items are rated on a 4-point Likert scale and can provide a measure of three subscales (Emotional Uncertainty, Desire for Control, Cognitive Uncertainty). As dependent variable, we considered both the three subscales scores and the total score.

The ASI-3 (Taylor et al., 2007; Petrocchi et al., 2015) is an 18-item, self-report measure developed to assess vulnerability to anxiety. Each item is rated on a 5-point Likert scale and higher scores reflect high level of anxiety. As dependent variables, we considered the Physical Concerns, Social Concerns and Cognitive Concerns subscales as well as the total score (sum of all the three subscales).

Finally, the EPQ-RS (Eysenck et al., 1985; Picconi et al., 2018) was used to assess personality characteristics of participants. EPQ-RS is a self-reported questionnaire with 48 dichotomous (yes, no) items, 12 for each of the traits of neuroticism, extraversion/intraversion and psychoticism, and 12 for the lie scale. As dependent variables, the scores of neuroticism, extraversion/intraversion, and psychoticism scales have been taken into consideration.

#### Experimental Tasks

##### Task 1. Recognition of Facial Expressions

Photographs of 10 actors (five males, five females) were taken from the Ekman and Friesen set of Pictures of Facial Affect (Ekman and Friesen, 1976; Ekman, 1993). Each model posed facial expressions corresponding to six basic emotions: happiness, sadness, anger, fear, disgust and surprise. The complete image set therefore included 60 stimuli (10 faces × 6 emotions). For each stimulus, subjects were required to name the expressed emotion selecting from six labels (happiness, sadness, anger, fear, disgust or surprise), and then to rate the emotion intensity expressed in the picture on a scale of 1–9 (1 = none, 5 = moderate, 9 = extreme).

##### Task 2. Evaluation of Emotionally Evocative Scenes

Stimuli consisted of complex pictures selected from the IAPS (Lang et al., 1997; Bradley and Lang, 2007). Stimulus selection was based on the results of a pilot study in which 80 university students assigned 200 IAPS scenes to one of six emotion labels (i.e., happiness, sadness, anger, fear, disgust and surprise) defined on the basis of emotional category data on IAPS (Lang et al., 1993; Davis et al., 1995; Bradley et al., 2001; Mikels et al., 2005). Different studies demonstrated that many of the IAPS images elicit multiple discrete emotions (Bradley et al., 2001; Mikels et al., 2005). For this reason, in the present experiment (see also Pistoia et al., 2010, 2015), we only used images classified by at least 70% of normal subjects consistently within one single emotional category. On this basis, we had to exclude stimuli intended to elicit surprise, because no item of this category reached the defined consistency level. Moreover, following Mikels et al.’s (2005) approach, we also excluded images being classified within two or more emotional categories. Therefore, the resulting image set included 30 images (6 items × 5 emotions), each consistently eliciting a single emotional category among the following emotions (Lang et al., 1993; Davis et al., 1995; Bradley et al., 2001; Mikels et al., 2005): happiness (scenes involving babies or sporting events), sadness (scenes of illness, cemeteries or funeral scenes), anger (scenes of human violence), fear (scenes of snakes or spiders), and disgust (scenes of rubbish or rats). Each stimulus was presented twice for a total of 60 items.

The subjects were first required to provide an emotional category rating by choosing among the five categories (happiness, sadness, anger, fear, or disgust) the one corresponding to the subjectively evoked emotion; the response was scored 1 if the subject selected the emotional category consistently evocated by the image, otherwise the response was scored 0. Then, the subjects had to rate how strong their own emotional response was on a scale of 1–9 (1 = not at all, 5 = moderately, 9 = extremely).

### Statistical Analysis

For the self-reported measures, a multivariate analysis of variance was performed with group and sex as independent variables and with scores on the self-reported measures as dependent variables. Level for statistical significance was set at *p* < 0.0033 according to Bonferroni’s correction for multiple comparisons. For the behavioral measures (recognition of facial expressions and evaluation of emotionally evocative scenes) a three-way mixed analysis of variance (ANOVA) was performed, with emotion as a within-subject factor and with group and sex as between-subject factors.

## Results

### Self-Reported Measures

The multivariate results showed significant effects for group (Pillai’s Trace = 0.241; Wilks’ Lambda = 0.759; *F*_(15,89)_ = 1.885; *p* = 0.035, partial eta squared = 0.241) and for sex (Pillai’s Trace = 0.313; Wilks’ Lambda = 0.687; *F*_(15,89)_ = 2.698; *p* = 0.45, partial eta squared = 0.230). The interaction between group and sex was not significant (*p* > 0.05).

For group (scores of the two groups on all the measures are reported in Table 1), there were significant univariate effects for: URS Total score (*F*_(1,103)_ = 9.079, *p* = 0.003, partial eta squared = 0.081), URS Emotional Uncertainty (*F*_(1,103)_ = 15.399, *p* = 0.0001, partial eta squared = 0.130), STAI-2 (*F*_(1,103)_ = 10.036, *p* = 0.002, partial eta squared = 0.089), ASI Total Score (*F*_(1,103)_ = 12.740, *p* = 0.001, partial eta squared = 0.110), ASI Cognitive Concerns (*F*_(1,103)_ = 10.944, *p* = 0.001, partial eta squared = 0.096), ASI Social Concerns (*F*_(1,103)_ = 11.777, *p* = 0.0001, partial eta squared = 0.118), and EPQ Neuroticism Scale (*F*_(1,103)_ = 10.260, *p* = 0.002, partial eta squared = 0.091). No other difference was significant at the Bonferroni corrected *p* value.

**Table 1:** Scores (mean and SD) of the two groups on the self-reported measures.

	Controls		Earthquake victims	
Measures	Mean	SD	Mean	SD
ISI	5.05	3.5	6.35	4
IUS-12	35.00	15.2	42.54	16.7
URS total score*	121.03	12.1	128.56	13.3
URS emotional uncertainty*	26.53	6.1	31.69	7.4
URS desire for control	47.22	7.2	46.13	7.7
URS cognitive uncertainty	47.41	8.5	51.10	8.9
STAI*	37.63	8.2	42.46	7.8
BDI	6.54	5.2	8.56	5.8
ASI-3 total score*	10.90	8.3	18.21	13.3
ASI-3 physical concerns	3.41	3.9	5.46	5.6
ASI-3 cognitive concerns*	4.05	3.6	6.77	5.1
ASI-3 social concerns*	3.44	3.4	6.25	5.1
EPQ-R Extraversion/Introversion	9.05	2.8	8.48	3.2
EPQ-R neuroticism*	4.46	2.7	6.33	3.3
EPQ-R psychoticism	3.22	1.9	2.67	1.5

ISI, Insomnia Severity Index; IUS-12, Tolerance of Uncertainty Scale Short Form; URS, Uncertainty Response Scale; STAI, State-Trait Anxiety Inventory; BDI, Beck Depression Inventory; ASI-3, Anxiety Sensitivity Index; EPQ-R, Eysenck Personality Questionnaire-Revised Short Form. *Significant at p ≤ 0.003.

For sex, there was only a significant univariate effect for IUS-12 (*F*_(1,103)_ = 15.322, *p* = 0.0001, partial eta squared = 0.129), with females scoring higher (mean = 44, SD = 16.1) than males (mean = 32.6, SD = 18.2). No other univariate effect was significant at the Bonferroni corrected *p* value.

### Experimental Tasks

#### Recognition of Facial Expressions

Percentages of correct responses are shown in Table 2. A three-way mixed ANOVA was carried out, with emotion (disgust, happiness, fear, anger, surprise and sadness) as a within-subject factor and with group and sex as between-subject factors. This showed a significant main effect of emotion (*F*_(5,515)_ = 36.897, *p* = 0.0001, partial eta squared = 0.264), with recognition of fear (0.60) being worse than all other emotions (disgust = 0.86; happiness = 0.99; anger = 0.89; surprise = 0.96; and sadness = 0.79). Results also showed significant main effects of group (*F*_(1,103)_ = 16.832, *p* = 0.0001, partial eta squared = 0.140), with overall accuracy being higher in earthquake victims (mean = 0.89, SD = 0.14) than in controls (mean = 0.81, SD = 0.12), and of sex (*F*_(1,103)_ = 4.208, *p* = 0.043, partial eta squared = 0.039), with females (mean = 0.87, SD = 0.13) being more accurate than males (mean = 0.83, SD = 0.14). No interaction was significant (all *p* > 0.05). *Post hoc* Bonferroni’s pairwise comparisons on the main effect of emotion demonstrated that recognition of happiness was significantly easier than all other emotions (all *p* < 0.001), followed by surprise (*p* = 0.01 vs. remaining emotions). No significant differences were detected between recognition of anger, sadness, or disgust (all *p* = 0.05), whereas recognition of fear was significantly worse than all other emotions (*p* = 0.005).

**Table 2:** Accuracy (mean and SD) and intensity rating on correctly recognized emotions (mean and SD) of the two groups on recognition of facial expressions task.

	Controls		Earthquake victims	
Accuracy	Mean	SD	Mean	SD
*Disgust*	0.84	0.2	0.88	0.3
*Happiness*	0.98	0.1	1.00	0
*Fear*	0.59	0.3	0.61	0.5
*Anger*	0.079	0.2	0.98	0.1
*Surprise*	0.93	0.2	0.99	0.2
*Sadness*	0.73	0.3	0.86	0.3
**Intensity rating**				
*Disgust*	6.20	1.1	6.59	0.9
*Happiness*	6.61	0.86	6.83	0.8
*Fear*	6.24	1.3	6.60	0.9
*Anger*	5.66	1.4	5.78	1.2
*Surprise*	6.38	0.9	6.55	1.1
*Sadness*	5.57	1.2	5.63	1.3

For intensity rating on correctly recognized emotions, we performed the same ANOVA as above, and results showed a significant main effect of emotion (*F*_(5,515)_ = 37.942, *p* = 0.0001, partial eta squared = 0.271), with happy faces (6.72) being rated more intense than all other emotion expressions (surprise = 6.45, fear = 6.41, disgust = 6.40, anger = 5.71, and sadness = 5.60). Results also showed a marginally significant main effect of sex (*F*_(1,103)_ = 3.939, *p* = 0.050, partial eta squared = 0.037), with females’ rating (mean = 6.38, SD = 0.11) being higher than males’ (mean = 6.05, SD = 0.11), whereas the main effect of group was not significant (*p* > 0.05). No interaction was significant (all *p* > 0.05). *Post hoc* Bonferroni’s pairwise comparisons on the main effect of emotion showed that rating of emotional intensity was significantly higher for happiness with respect to all the other emotions (*p* = 0.030), followed by surprise, fear and disgust which were rated significantly more intense than anger and sadness (*p* = 0.0001). Ratings of surprise, fear and disgust did not significantly differ from each other (*p* > 0.05).

#### Evaluation of Emotionally Evocative Scenes

Emotional category ratings are shown in Table 3. A three-way mixed ANOVA was carried out, with emotion (disgust, happiness, fear, anger and sadness) as a within-subject factor and group as a between-subject factor. This did not reveal significant main effects of group or sex (both *p* > 0.05) but showed a significant main effect of emotion (*F*_(4,412)_ = 39.545, *p* = 0.0001, partial eta squared = 0.277), with the rating of scenes evocating anger (0.59) being more difficult as compared to the other emotions (fear = 0.73; disgust = 0.78; sadness = 0.89; happiness = 0.90). *Post hoc* Bonferroni’s pairwise comparisons demonstrated that the rating of scenes evocating anger was significantly more difficult than that of the other emotions (all *p < 0.005)*. No significant differences were detected between the rating of scenes evocating anger and disgust and between scenes eliciting sadness and happiness (all *p* > 0.05); the rating of scenes eliciting sadness and happiness was significantly easier that that concerning the other emotions (all *p* < 0.0001).

**Table 3:** Emotional category rating (mean and SD) and intensity rating of affective scenes (mean and SD) of the two groups on judgment of emotionally evocative scenes task.

	Controls		Earthquake victims	
	Mean	SD	Mean	SD
**Emotional category rating**				
*Disgust*	0.80	0.2	0.77	0.2
*Happiness*	0.91	0.3	0.90	0.2
*Fear*	0.73	0.2	0.73	0.3
*Anger*	0.62	0.3	0.56	0.3
*Sadness*	0.87	0.4	0.91	0.1
**Intensity rating**				
*Disgust*	6.05	1.5	6.06	1.6
*Happiness*	6.46	1.4	6.87	1.4
*Fear*	6.16	1.8	6.43	1.4
*Anger*	7.44	1.3	7.79	1.3
*Sadness*	6.27	1.6	6.35	1.4

Intensity ratings on correct responses were analyzed by the same three-way mixed ANOVA as above, and results showed a significant main effect of sex (*F*_(1,103)_ = 10.362, *p* = 0.002, partial eta squared = 0.091), with females’ rating (mean = 6.89, SD = 0.14) being more higher than males’ (mean = 6.24, SD = 0.15), whereas the main effect of group was not significant (*p* > 0.05). Moreover, results showed a significant effect of emotion (*F*_(4,412)_ = 37.678, *p* = 0.0001, partial eta squared = 0.229), with scenes evocating anger (7.56) being rated as more intense than all other scenes (happiness = 6.65, fear = 6.30, sadness = 6.30, and disgust = 6.03). *Post hoc* Bonferroni’s pairwise comparisons on the main effect of emotion showed that the rating of emotional intensity was significantly higher for scenes evocating anger with respect to all the other scenes (*p* = 0.0001), whereas the ratings of scenes evocating happiness, fear, sadness and disgust did not significantly differ between each other (*p > 0.05)*.

## Discussion

The main findings of the present study demonstrated a general increase of anxiety and anticipation of threats, as well as a tendency toward sleep problems in earthquake victims, which is consistent with previous literature on the mental health and psychological problems of earthquake victims (Maltais et al., 2001; Tempesta et al., 2013; Ferrara et al., 2016; Bianchini et al., 2017; Labra et al., 2017). Importantly, moreover, behavioral experiments demonstrated significantly higher accuracy of the earthquake-exposed group in recognizing facial expressions as compared to the control group. This was notwithstanding a comparable capacity to evaluate own emotional response to affective scenes. Considering this combination of results, we would suggest that exposure to earthquake selectively increased vigilance for threat detection leading earthquake victims to systematically pay attention to stimuli signaling potential threats, as in the case of emotional facial expressions. Our interpretation is consistent with that recently provided by Bell et al. (2017), who studied individuals exposed to the 2010–2011 Canterbury (new Zealand) earthquake. They found that both individuals with PTSD and earthquake-exposed individuals without PTSD had increased accuracy in recognition of emotional facial expressions as compared to a non-exposed control group. The authors suggested that the earthquake-exposure affected the recognition of facial expressions, independently from the development of a clinical psychopathological disease, by increasing the sensitivity to threat-related stimuli.

Hypervigilance towards environmental stimuli, which signal potential sources of threat, can be an adaptive mechanism following the exposure to a traumatic event as it can be advantageous to efficiently check the surrounding context in order to detect an upcoming threatening event. In this respect, being accurate in processing the others’ facial expressions may be particularly useful, because such stimuli provide highly relevant social information and play a key role in emotional appraisal of self-relevant threats. However, it is worth noting that the increased accuracy of earthquake-exposed participants was not restricted to threat-related expressions (such as angry and fearful faces). This result is again consistent with the findings of Bell et al. (2017) who suggested that this trend could be related to the prolonged exposure to aftershocks in the earthquake-exposed individuals. As an alternative explanation, we suggest that the development of a hypersensitivity to emotional facial expressions, irrespective of the specific emotional category, could represent an effective way to rapidly detect the presence of self-relevant threatening events in the surroundings (Sander et al., 2003). Disentangling between these two alternative interpretations was outside the main aims of the present study, but this issue merits a direct investigation.

The novel result of the present study was that the higher accuracy in emotional faces recognition was a specific emotional response to the traumatic event rather than the expression of a general, heightened sensitivity to affective information. This was demonstrated by the findings that earthquake victims did not show any difference from non-exposed participants in evaluating the nature of their own reaction to the presentation of affective scenes not involving faces. A dissociation between explicit recognition of emotional facial expressions and evaluation of affective scenes has been previously reported by our group in studies on clinical populations with specific neurological disorders involving damage of motor and of sensory pathways, respectively (Pistoia et al., 2010, 2015). The present study could demonstrate for the first time that such a selective effect on processing of emotional faces can also be the result of exposure to a stressful, traumatic event and can be related to the development of specific expertise allowing earthquake victims to effectively detect threats in the surrounding environment.

Bianchini et al. (2017) investigated the relationship between the presence of anxiety and depressive symptoms following the trauma and the implementation of coping strategies (post-traumatic growth) within a university student community exposed to the 2009 L’Aquila earthquake, 2 years after the traumatic experience. Results demonstrated that 13.3% of the sample reported anxiety and about 60% showed variable levels of depression, with moderate levels of depression being predictive of post-traumatic growth. The authors suggested that some psychopathological conditions with a typically negative connotation, such as depression, might promote the development of a positive post-traumatic response, likely through the implementation of metacognitive skills that, in turn, can favor positive and functional coping strategies. Within this interpretative framework, greater accuracy in recognizing facial expressions could represent a “positive” emotional response of earthquake victims, who are forced to constantly deal with emotional signals of threat. In recent years a number of behavioral and neuroimaging studies have demonstrated that experience can shape the persons’ ability to analyze and respond to specific categories of stimuli. For instance, while observing needles being inserted into others’ body parts, physicians who are expert in acupuncture showed a specific brain activation involving areas devoted to the understanding of others’ mind and to the regulation of affective responses (Cheng et al., 2007). Accordingly, Conson et al. (2013) demonstrated that professional actors with specific training in voluntary activation of mimicry to reproduce character’s emotions were better at the explicit recognition of facial expressions than both non-professionals and professional actors trained to infer other’s inner states from reading the emotional context. The authors argued that experience can selectively influence explicit recognition of others’ facial expressions, depending on the kind of “emotional expertise” acquired. The present study suggests that such expertise in explicitly decoding others’ facial expressions can be the result of trauma exposure. However, it seems to be a maladaptive rather than a functional emotional response to trauma, since the earthquake victims showed a higher degree of anxiety, insomnia and threat anticipation. The present results can therefore be best accounted for by a hypervigilance toward threat-related stimuli, as consistently found not only in people affected by different anxiety disorders (e.g., generalized anxiety disorder, specific phobias, social phobia or PTSD), but also in nonclinical individuals reporting high levels of anxiety (Dalgleish et al., 2003; Bar-Haim et al., 2007). Indeed, although hypervigilance to threats facilitates the detection of danger in the environment and helps the organism to respond effectively to threatening situations, it plays a central role in the etiology and maintenance of anxiety disorders (Beck, 1976; Eysenck, 1992; Mathews and MacLeod, 2002).

One possible limitation of the study lies in the fact that we tested the processing of emotional faces by employing highly prototypical stimuli displaying emotional faces with a straight gaze, as previously made in most of the studies addressing the same issue. However, one should take into account that such a laboratory setting cannot completely simulate the action of processing emotional facial expressions in real-life situations (Hess and Blairy, 2001). In this respect, one could manipulate both emotional category and direction of eye gaze (direct vs. averted) of the facial expression, thus developing more ecologically valid, self-relevant threatening stimuli (Sander et al., 2003; Ponari et al., 2013).

From a translational point of view, the present findings could pave the way for the implementation of specific preventive and treatment options for earthquake-exposed people by exploiting available techniques of cognitive biases modification, such as attentional bias modification (MacLeod and Clarke, 2013). Attentional bias modification is an emerging treatment approach designed to modify the patterns of attentional selectivity favoring the processing of threatening information. Importantly here, several studies with both clinical and non-clinical populations have demonstrated that this technique can reduce emotional vulnerability (Clarke et al., 2014). We did not use a classical attentional bias task (MacLeod et al., 1986; Bar-Haim et al., 2007) to demonstrate a condition of hypervigilance toward emotional faces in earthquake victims, but our findings suggest that the implementation of an attentional bias modification paradigm might help to reduce the pattern of anxiety responses in earthquake-exposed people. It might also prevent the possible development of clear psychopathological disorders in victims showing subclinical conditions.

In conclusion, the present study indicates a greater accuracy of earthquake victims in recognizing facial expressions, despite the lack of difference from controls in evaluating emotionally evocative scenes. A possible explanation for this effect is that trauma exposure increases threat detection in earthquake victims, leading them to systematically pay much more attention to every kind of potential sign of threat. This may lead people exposed to trauma to progressively develop specific, “emotional expertise.” Further studies are necessary to confirm our findings and to implement preventive and treatment approaches to boost resilience and encourage coping strategies in exposed populations.

## Author Contributions

FP, MC, AC, MGD, AS, GC and SS provided their substantial contributions to the conception of the work, the acquisition, analysis and interpretation of data and to the writing of the draft manuscript.

## Conflict of Interest Statement

The authors declare that the research was conducted in the absence of any commercial or financial relationships that could be construed as a potential conflict of interest.
